# Habitat preferences, estimated abundance and behavior of tree hyrax (*Dendrohyrax* sp.) in fragmented montane forests of Taita Hills, Kenya

**DOI:** 10.1038/s41598-022-10235-7

**Published:** 2022-04-15

**Authors:** Hanna Rosti, Janne Heiskanen, John Loehr, Henry Pihlström, Simon Bearder, Lucas Mwangala, Marianne Maghenda, Petri Pellikka, Jouko Rikkinen

**Affiliations:** 1 Finnish Museum of Natural History, University of Helsinki, P.O. Box 7, 00014 Helsinki, Finland; 2 Organismal and Evolutionary Biology Research Programme, Faculty of Biological and Environmental Sciences, University of Helsinki, P. O. Box 65, 00014 Helsinki, Finland; 3 Department of Geosciences and Geography, Faculty of Science, University of Helsinki, P.O. Box 64, 00014 Helsinki, Finland; 4 Lammi Biological Station, University of Helsinki, Pääjärventie 320, 16900 Lammi, Finland; 5 Molecular and Integrative Biosciences Research Programme, Faculty of Biological and Environmental Sciences, University of Helsinki, P. O. Box 65, 00014 Helsinki, Finland; 6 Nocturnal Primate Research Group, Oxford Brookes University, Oxford, OX3 0BP UK; 7 Programme and Planning, Academic Research and Outreach Division, TAITAGIS, Taita Taveta University (TTU), P. O. Box 635-80300, Voi, Kenya; 8 Department of Agricultural Sciences, School of Agriculture Earth and Environment Sciences, TAITAGIS, Taita Taveta University (TTU), P. O. Box 635-80300, Voi, Kenya; 9 Taita Research Station of University of Helsinki, P.O. Box 1156, Wundanyi, 80304 Taita Taveta Kenya

**Keywords:** Behavioural ecology, Biodiversity, Conservation biology, Tropical ecology, Animal behaviour

## Abstract

We studied a previously almost unknown nocturnal mammal, an apparently undescribed species of tree hyrax (*Dendrohyrax* sp.) in the moist montane forests of Taita Hills, Kenya. We used thermal imaging to locate tree hyraxes, observe their behavior, and to identify woody plants most frequently visited by the selective browsers. We also documented acoustic behavior in forest fragments of different sizes. Data on calling type and frequency were analyzed together with lidar data to estimate population densities and to identify forest stand characteristics associated with large populations. Viable populations were found only in the largest forest fragments (> 90 ha), where tree hyraxes preferred most pristine forest stands with high, multilayered canopies. The estimated population sizes in smaller forest fragments were very limited, and hyraxes were heard to call only during late night and early morning hours, presumably in order to avoid detection. While we frequently recorded tree hyrax songs in the largest forest fragments, we almost never heard songs in the small ones. All remaining subpopulations of the Taita tree hyrax are under threat of human disturbance and further habitat deterioration. Conservation efforts should include protection of all remaining habitat patches, but also reforestation of former habitat is urgently needed.

## Introduction

Over much of the tropics, rapid deforestation has led to highly fragmented forest landscapes^1^. Habitat fragmentation influences species richness and persistence, and often results in extinction debt^2^. Negative consequences in spatially restricted biodiversity hotspot areas, like the Eastern Arc Mountains of East Africa, have been particularly severe. More than 80% of the indigenous moist montane forests of the Eastern Arc Mountains have been cleared, and 25% of them have been lost since 1955^3^. This development has most likely led to extinction debt as mammal species in small and fragmented habitats tend to be close to their extinction threshold^4^. For example, in Thailand most mammal species of small forest fragments (10–56 ha) went extinct within 25 years^5^.

**Figure 1:**
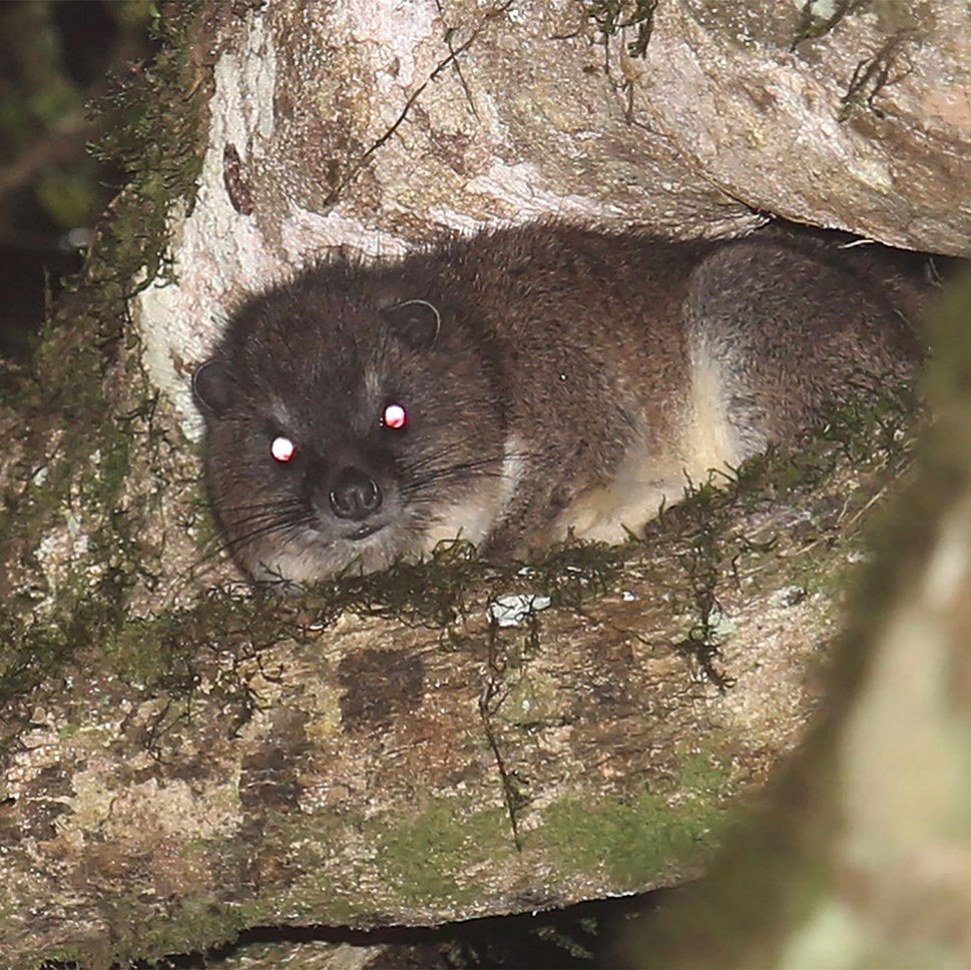
*Dendrohyrax* sp. resting on a branch of *Strombosia scheffleri* (Photographed by Hanna Rosti in Mbololo Forest 2021).

The Taita Hills in southeastern Kenya represent the northernmost extension of the Eastern Arc Mountains, and the moist montane forests of the mountains, together with the coastal forests of Kenya and Tanzania, represent a hotspot of biodiversity with many endemic animal and plant species^6,7^. The Taita Hills fauna includes tree hyraxes (*Dendrohyrax*), which are medium-sized herbivorous afrotherian mammals (Fig. 1). Across Africa, four species, western tree hyrax *D. dorsalis*, eastern tree hyrax *D. validus*, southern tree hyrax *D. arboreus*, and Benin tree hyrax *D. interfluvialis*, are currently recognized; however, additional undescribed species probably await scientific discovery^8–12^. Previously the Taita Hills tree hyraxes were supposed to represent eastern tree hyrax *D. validus*^13^. This species was originally described by True^14,15^ from Mt Kilimanjaro, Tanzania, and Taveta, a border town in present Kenya, close to Kilimanjaro. However, the Taita Hills tree hyraxes differ from typical *D. validus* in many respects. For example, their vocalizations include distinctive songs, and their most frequently used call is the unique and earsplitting “strangled thwack”^12^. Rosti et al.^12^ suggested that the Taita Hills hyraxes may belong to a previously unrecognized taxon and here, we provisionally refer to these animals as “*Dendrohyrax* sp.”.

Many aspects of tree hyrax ecology remain very poorly known. They are known to mainly feed on leaves of woody plants^10,16,17^. Tree hyraxes thus depend on trees for food, but they also utilize den trees for shelter, and dense canopy also allows easy movement between trees^10,16,18–22^. Tree hyraxes are thought to be mainly solitary animals that maintain social relationships by calling and scent-marking signals^16^. They are highly vocal and calling seems to be their primary means of communication^10,12,13,23^. Tree hyraxes in Taita Hills also sing^12^ in a similar manner as rock hyraxes^24–26^. They call most actively immediately after dusk and before dawn ^23^ and urinate and defecate from particular places up in the trees^10,16^.

Lawes et al. ^27^ studied consequences of forest fragmentation to *Dendrohyrax arboreus* populations in South Africa*.* They concluded that close to larger forests tree hyraxes were able to also survive in small forest patches (< 6 ha), even under moderate human disturbance. However, in small forest patches located more than 1.5 km from larger forests, the probability of finding tree hyraxes was almost zero. Size of tree hyrax populations is difficult to estimate, as sightings of animals are rare. Kundaeli^16^ estimated tree hyrax population density based on defecation sites and concluded that tree hyraxes are most abundant in areas where logging intensity is low. Topp-Jørgensen et al.^28^ estimated tree hyrax relative density using circular plots with a 50 m radius and calculated the number of calling individuals. They found that abundance of tree hyraxes correlated negatively with open-canopy structure and hunting.

Calling frequency of tree hyraxes provides useful data that can be used for estimating relative population abundance^28^. Generally, acoustic recordings play an increasingly important role in monitoring biodiversity in many types of environments^29,30^. Passive recorders allow the collection of a large body of data simultaneously from several locations and over an extended time-period. They are particularly useful when visual surveys are difficult, such as with nocturnal animals, if only the animals produce readily detectable sounds. Passive recorders have previously been used to produce population size estimates for birds^31^ and bats, and they have also been used in combination with occupancy modelling^32^. Airborne lidar can be used to obtain accurate data on many different attributes of forest structure, which are of value for studies of arboreal mammals^33–35^. Airborne lidar systems send laser pulses that are able to penetrate the forest canopy all the way to the ground and can hence provide information on both canopy height and the vertical and horizontal distribution of plant biomass^36^.

**Figure 2:**
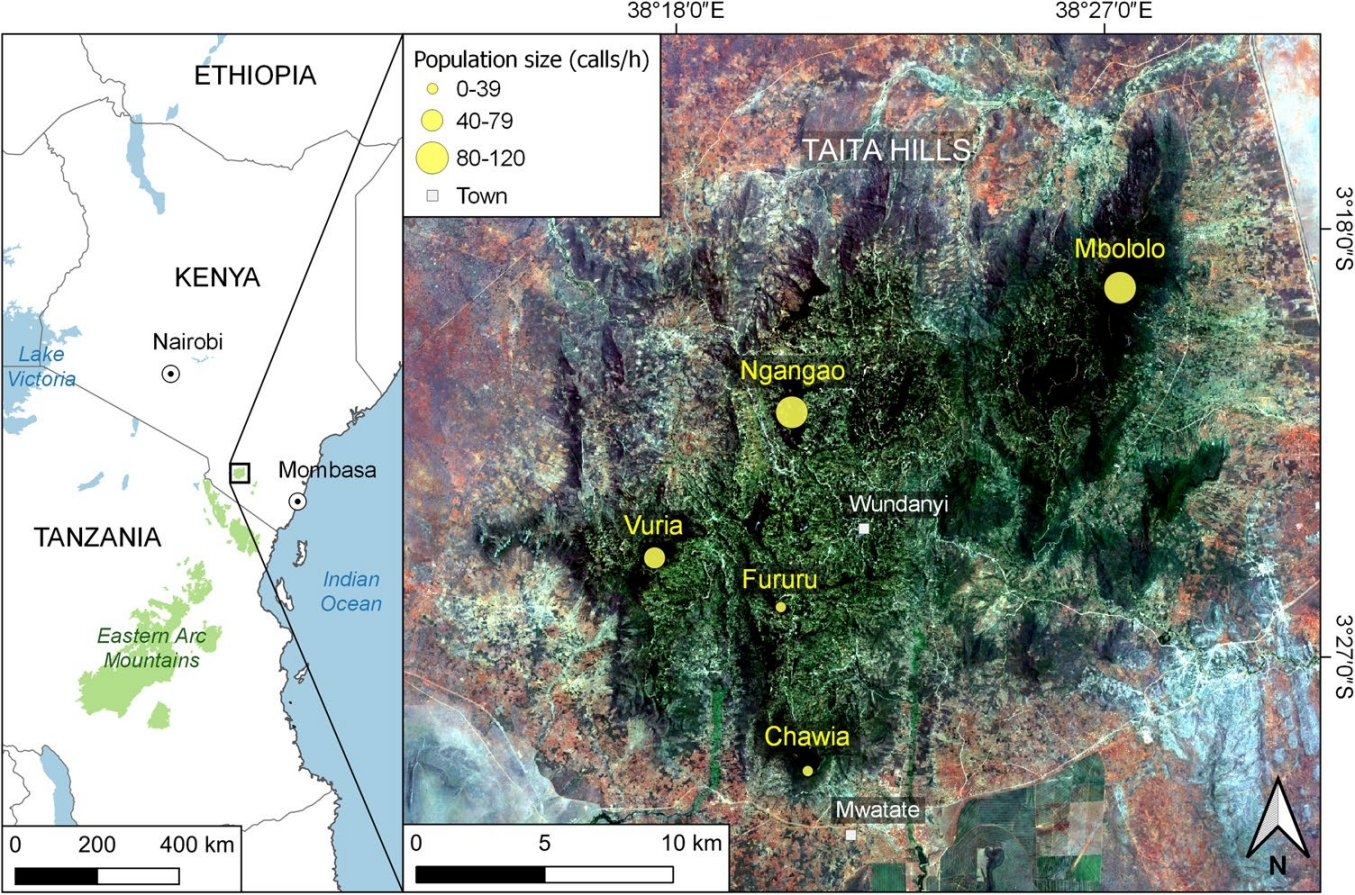
Location of study sites in the Taita Hills. Study forests and AudioMoth recording sites are in yellow. Circle size corresponds with the estimated size of *Dendrohyrax* populations in the forests. Wundanyi is the largest town in the region. The map was created by QGIS version 3.16.16, https://qgis.org/en/site/.

The Taita Hills consist of a series of mountain ridges reaching 2208 m a.s.l. at Vuria, the highest peak of Dabida (Fig. 2). The highland consists of three closely situated mountain massifs (Dabida, Mbololo, Sagalla) and Mount Kasigau, located further away, all rising abruptly form the surrounding semiarid plains. The upper elevation slopes of the Taita Hills were formerly covered by moist montane forest^37,38^. Because of rapid population growth and intensified land use, only small fragments of the original forest cover are now left, and the twelve remaining fragments of indigenous forest cover a combined total area of only about 7 km^2^ ^39^. They represent the last refugia for a wide diversity of species, including many endemic taxa, that depend on moist indigenous forests^6,40–42^.

In this study, we combined the use of a thermal imaging camera, passive audio recorders, and airborne lidar data analysis to study tree hyraxes in the highly fragmented forest landscape of the Taita Hills. We collected most of our data from five forest fragments: Mbololo (185 ha), Ngangao (120 ha), Vuria (96 ha), Chawia (85 ha), and Fururu (8 ha), which represent the largest remaining fragments of indigenous moist montane forest in the Taita Hills (Fig. 2, Table S1). We made casual observations in two more distantly located montane forests and in a wide variety of marginal habitats within the matrix of agricultural land and plantation forests surrounding the main forest fragments. With thermal imaging, we could observe tree hyrax behavior without disturbing the animals. It also allowed us to identify tree species and individual trees that the animals most frequently visited. We used passive audio recorders to document and to analyze acoustic behavior at several sites in five forest fragments and we analyzed the data on calling frequency together with lidar data to estimate population densities (calling frequency was expected to correlate with population density) and to identify forest stand characteristics associated with highest tree hyrax population densities.

## Results

### Distribution and habitat preferences

Viable tree hyrax populations exist in Mbololo, Ngangao and Vuria, the three largest remaining fragments of indigenous montane forest in the Taita Hills (Fig. 2). Chawia and Fururu support smaller populations, and some individuals or small groups also exist outside the major forest fragments, for example in tiny pockets of indigenous forest vegetation around Yale Hill. We found no evidence of tree hyraxes in the indigenous forests of Mount Kasigau and Sagalla Hill.

Tree hyraxes were seen at different heights on many species of indigenous trees, but they obviously preferred climber covered trunks and multilayered canopies of large upper canopy trees (Table S2). They were easiest to spot during windy nights, probably because the animals tended to descend to the lower canopy layers under such conditions. Tree hyraxes were most frequently observed resting on large vertical branches or on tilted trunks and/or among tangled growths of woody climbers. Tree hyraxes are surprisingly agile and commonly use lianas to move from one tree to another. In Ngangao Forest, most sightings were made from *Macaranga capensis* (Euphorbiaceae)*, Tabernaemontana stapfiana* (Apocynaceae), and *Albizia gummifera* (Fabaceae), and we observed tree hyraxes feeding on leaves of all these species. While foraged trees commonly had leaves at different stages of development, we repeatedly saw tree hyraxes reaching for the youngest leaves at the tips of growing branches.

In Ngangao Forest, about 60% of all tree hyrax sightings were from trees that supported woody climbers (Table S2). The lianas were used as a food source, for movement, and for resting and shelter. Tree hyraxes were often observed feeding on the leaves of *Dichapetalum eickii* (Dichapetalaceae). Other woody climber species included for example *Dalbergia lactea* (Fabaceae), *Clerodendron capitatum* (Lamiaceae), *Ficus thonningii* (Moraceae), *Hippocratea goetzei* (Celastraceae), and *Rourea thomsonii* (Connaraceae). In Mbololo Forest, tree hyraxes were mostly seen in the canopy of *Macaranga capensis* (29%), *Strombozia scheffelerii* (18%), *Tabernaemontana stapfiana* (18%), and *Newtonia buchananii* (12%).

In Taita Hills, tree hyraxes defecate from trees to the forest floor. We found fecal pellets of different ages under occupied trees, but they tend to decompose quickly. We have found only three sites from Ngangao and Mbololo that can be described as dung middens.

### Calling behavior

Tree hyraxes may call from all canopy heights, both while stationary and when on the move. Directing a red flashlight beam towards a tree hyrax would silence it and usually the animal would soon move away from the light. However, a thermal imaging camera did not affect their calling or other behavior. Taita tree hyraxes only call during the night. They typically emit their first calls soon after dusk (at 18.45–19:00 h) and calling ends just before dawn (before 06:00 h) in the morning. Tree hyraxes seem to be much less vocal during the height of the dry season in September, when many trees shed part of their foliage. Perhaps the animals save energy during the dry season, and thus call less.

**Figure 3:**
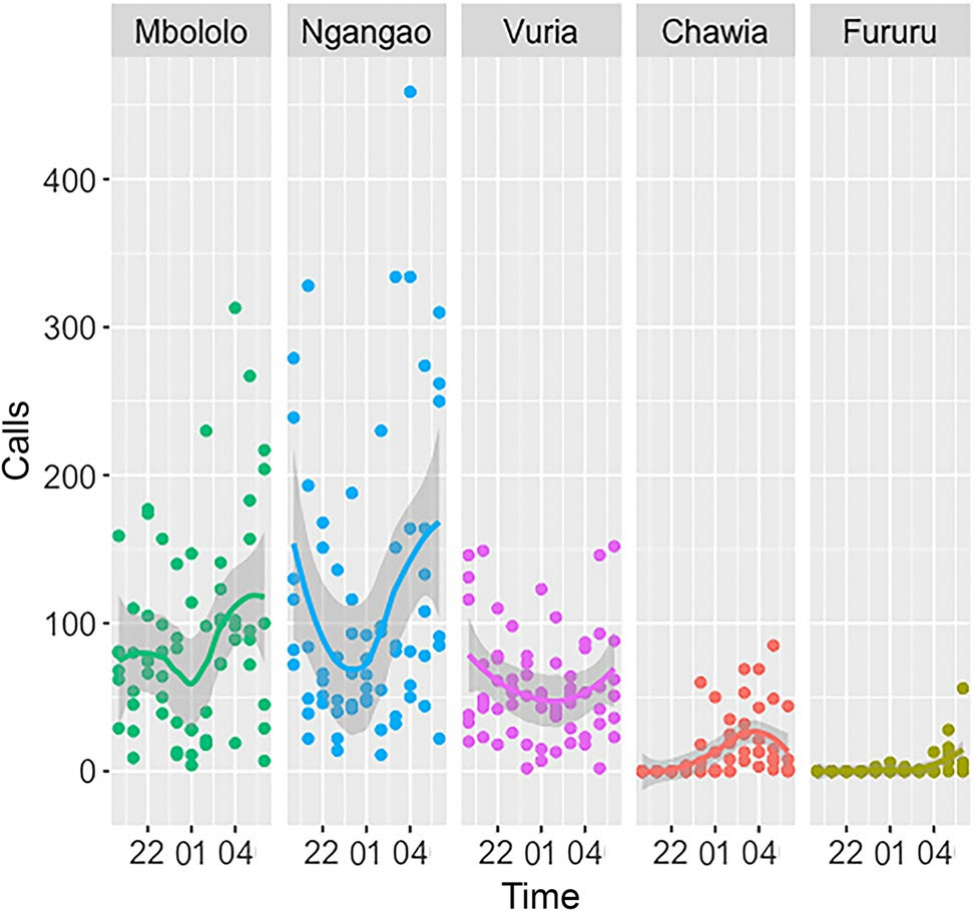
Number of calls at different hour of the night in five forest fragments of the Taita Hills (for more information, see Table S3). The image was created with software R version 3.6.3, https://www.r-project.org/”, and modified with Adobe Photoshop, version 22.3, https://www.adobe.com/.

The average number of calls given by tree hyraxes for each hour between 19:00 and 06:00 h varied between 3 (Fururu) and 116 (Ngangao) (Fig. 3, Table S3). 25% of all observation hours had no calls, and all such hours were recorded from Chawia and Fururu. The highest recorded calling rate was from Ngangao. The maximum for Mbololo was lower, but the extremely steep mountain slopes of this forest prevented us from placing AudioMoths at some optimal sites. Vuria, which supports a mixture of remnant indigenous forest patches and plantation trees around the peak, also has a relatively dense and vocal population of tree hyraxes. The tree hyraxes in Chawia and Fururu start calling much later than the animals at the other study sites, with the first calls typically being emitted around midnight, whereas in Mbololo, Ngangao and Vuria the animals start calling soon after dusk. Calling frequency was also very low, compared to the larger forest fragments; during most hours, the tree hyraxes were not calling at all (Fig. 3, Table S3).

Of all the analyzed hours (n = 319), 37 contained tree hyrax songs (Table S3). Singing was most frequent in Mbololo and Ngangao, where more than 20% of hours contained songs. In Vuria, singing was less frequent, with 9% of singing hours. In both Chawia and Fururu, tree hyraxes were observed singing only once during the recording period. Tree hyrax songs are commonly duets, but sometimes three or four individuals join in. If two or more animals take part, the singing bouts tend to last longer. Singing may continue from less than 1 min to up to 23 min (Audio File S1).

According to our observations, individual tree hyraxes seem to have distinctive calls, and others probably recognize who is calling. The animals of one group do not usually respond to calls coming from other groups. We repeatedly observed tree hyraxes feeding and resting close to calling animals, and completely ignoring them. 75% of calling sequences in Taita Hills are countercalls between 2 and 6 animals, typically 4^12^. Some animals from the group are counter calling, while others keep feeding or resting. We saw 2–3 hyraxes peacefully together in the same tree nine times, and several times, we witnessed animals resting together side by side.

### Statistical modelling of calling frequency and singing

ZINB GLMM analysis model (calls-size*time + height + cc10 + (site)) on all forests indicated that “forest size” in interaction with “time” were statistically significant variables to explain calling frequency for each hour (Table 1a, Fig. S1a). Canopy height (zq99) and canopy cover at 10 m height (cc10) were not statistically significant, but they were kept in the model, because in stepwise model selection as judged by Akaike’s Information Criterion (AIC), this improved the model (Table 1b, Fig. S1b). Model validation indicated no problems. The model explained, according to conditional R^2^, 65% of the variance, and the R^2^ marginal, without random effects, was 44% of the variance. The intra-class coefficient ICC was 0.37.

**Table 1:** Results of statistical analyses.

(a) Estimated regression parameters, standard errors (SE), z-values and P-values for the ZINB GLMM with model: calls—size*time + zq99 + cc10 with all the forests of Taita Hills				
	Estimate	SE	z-value	P-value
Intercept	4.488580	0.18664	24.049	< 2E − 16
Size	− 0.407968	0.25540	− 1.597	0.00180
zq99	0.317512	0.152841	2.077	0.16564
cc10	0.334376	0.242165	1.381	0.46496

(b) AIC and ∆AIC with step1 function with the model calls—size*time + zq99 + cc10 with all the forests of Taita Hills				
	Df	AIC	∆AIC	
None		2805.7	0	
Size*time	11	2891.3	85.6	
cc10	2	2902.2	96.5	
zq99	2	2924	118.3	

(c) Estimated regression parameters, standard errors (SE), z-values and P-values for the NB GLMM with model: calls—size*time + zq99 + cc10 from Mbololo, Ngangao and Vuria Forests				
	Estimate	SE	z-value	P-value
Intercept	4.498331	0.181644	24.765	< 2E − 16
zq99	0.339377	0.147054	2.308	0.03776
Size	− 0.375196	0.241463	− 1.554	0.11019
cc10	− 0.274987	0.228903	1.201	0.16735

(d) AIC and ∆AIC with step1 function with the model calls—size*time + zq99 + cc10 from NB GLMM analysis				
	Df	AIC	∆AIC	
None		2102.1	0	
Size*time	11	2109.9	7.3	
cc10	2	2121.0	18.9	
zq99	2	2147.4	45.4	

(e) Estimated regression parameters, standard errors (SE), z-values and P-values for the Bernoulli GLMM model of singing probability with forest size as the only variable				
	Estimate	SE	z-value	P-value
Intercept	− 2.3104	0.2481	− 9.313	< 2E − 16
Size	0.8513	0.2210	3.853	0.000117

A similar model for the three largest forest fragments (calls-size*time + height + cc10 + (site)) in NB GLMM analysis (all > 90 ha and without 0 h) indicated that canopy height (zq99) was the only statistically significant variable to explain calling frequency for each hour (Table 1c). In accordance with AIC, size*time, and cc10 were kept in the model (Table 1d). Model validation indicated no problems. The model explained, according to conditional R^2^, 57.5% of the variance, and the R^2^ marginal, without random effects, was 39.5% of the variance. Intra-class coefficient ICC was 0.27.

Bernoulli GLMM analysis of the effect of forest fragment size on tree hyrax singing indicated that “forest size” was a highly significant covariate in tree hyrax singing probability (P-value < 0.0001) (Table 1e, Fig. S1c). The model explained, according to conditional R^2^, 19% of the variance, and the R^2^ marginal, without random effects, was 18% of the variance. Intra-class coefficient ICC was 0.017.

## Discussion

Most human-modified landscapes consist of a mosaic of environments with different degrees of suitability for the existence and composition of species^43^. *Dendrohyrax validus* is currently classified as Near Threatened (NT) by the IUCN^44^, but knowledge about tree hyraxes in general is still very limited, including their taxonomic diversity^8,10,12^.

Based on observations and data collection during three years of fieldwork, we estimate that current tree hyrax density in the indigenous and mixed moist montane forests of Taita Hills is 0–13 animals per hectare of remaining forest. This estimation is based on the size of the remaining forest fragments and estimated group size. Estimated territory of each group is 2 ha. There are also unoccupied patches between calling groups where solitary individuals may be encountered. Our estimate is well in line with that of Topp-Jørgensen et al.^28^ who estimated hyrax population density in the Udzungwa Mountains, Tanzania, based on circular plots, midden counts, and daytime transect counts. Their result was 17.3 individuals/ha in an undisturbed forest, 12.1 individuals/ha in a lightly disturbed forest, and zero in a formerly logged forest with intense hunting pressure. We estimate that Mbololo Forest currently has about 1000–1900 tree hyrax individuals, Ngangao 400–750, Vuria 255–530, Chawia 20–40, and Fururu 10–20. Small patches of indigenous forest vegetation, including sacred sites and traditionally protected community forests^45^, also have some tree hyraxes, but not many, as the combined area of dozens of such forests is only about 1 km^2^. Additionally, some tree hyrax groups, and individuals persist outside indigenous forest fragments. Taken together, there may currently be only 1700–4000 tree hyrax individuals in the Taita Hills region.

We expected tree hyrax population density (as estimated by calling frequency) to decrease with decreasing forest size, and indeed this was the case when all forests were analyzed together. This indicates that in Taita Hills, where the remaining forests are very small and fragmented, tree hyrax populations start to deteriorate after the size of the forest fragment falls to < 90 ha. In the analysis with only the three largest forest fragments, canopy height was more important than forest size. Thus, while tree hyraxes clearly prefer habitats with tall and massive trees, when the size of a forest decreases, the effect of forest size per se becomes more important, presumably because human disturbance tends to intensify with decreasing habitat size.

Lawes et al.^27^ found that the minimum area for *Dendrohyrax arboreus* in South Africa in their metapopulation model was 6 ha. While some tree hyraxes currently exist in tiny forest fragments (< 2 ha) in the Taita Hills, without an ability to disperse to other habitat patches, these populations are subjected to many threats, including inbreeding. In South Africa, *D. arboreus* was able to colonize forest fragments that were 0.9 km from the main area, but unable to colonize patches that were 1.5 km away^27^. All the remaining forest fragments in Taita Hills are located near mountaintops that are several kilometers apart, and tree hyraxes are probably unable to cross this distance through densely populated lower-elevation areas.

All the tree species listed in Table S2 are common and often abundant in moist indigenous forests of the Taita Hills^37,46^. Some of the same tree species were also reported from Mau Forest in the Rift Valley of Kenya, where Milner^18^ studied tree hyraxes. Kundaeli^16^ studied *Dendrohyrax validus* in montane forests of Mount Kilimanjaro and found them to prefer *Ocotea usambarensis, Schefflera volkensii*, *Podocarpus latifolia,* and *Nuxia congesta.* All these species also occur in the Taita Hills, but *Ocotea* and *Podocarpus* have been exploited in the past to such a degree that large individuals are now rare or locally extinct in all remaining forests. In Ngangao Forest, which was also subjected to selective felling in the past, the tallest trees (> 50 m) presently represent individuals of *Newtonia buchananii* and *Pouteria adolfi-friedericii*. The massive crowns of the upper canopy emergents are likely to provide a refuge for tree hyraxes, but unfortunately, this interesting niche was out of practical reach of our thermal imaging camera.

In addition to large den trees^10,16,19,22,28,29^, our observations with thermal imaging camera indicated that woody climbers are very important for tree hyraxes, both as a food source and in providing shelter and resting places. For example, *Dichapetalum eichii* often forms extensive and tightly tangled nets around massive tree trunks, offering tree hyraxes perfect hideouts, where they can only be spotted with the help of a thermal imaging camera. All this supports the conclusion of Kundaeli^16^, who suggested that access to cavity-bearing trees was a primary factor that limited population density of tree hyraxes. As in Kilimanjaro, limitations in food availability may not be as decisive, as most tree species of moist montane forests retain most of their foliage throughout the year. In the Taita Hills tree hyraxes were sometimes spotted resting on vertical branches of Mexican cypress (*Cupressus lusitanica*), which is an introduced plantation species. Our general impression is that forest stands dominated by indigenous trees mixed with moderate amounts of mixed Mexican cypress or Mexican weeping pine (*Pinus patula*) can represent suitable habitats for tree hyraxes, if adequate shelter exists in the form of e.g. large den trees, woody climbers or within dry leaves of African palm (*Phoenix reclinata*). In the Udzungwa Mountains in Tanzania, tree hyrax middens may cover areas of several square meters and imbue adjacent tree trunks and the ground with a strong-smelling odor and a thick, tarmac-like coating^28^. In Taita Hills, we found only a few middens, which might be partly due to rapid decomposition of dung, but also the high level of human disturbance, which may prevent the animals from establishing regular middens at most forest sites.

Poaching has been recognized as a major threat for tree hyraxes on Mount Kilimanjaro^16^ and on other Eastern Arc Mountains in Tanzania^23,28^. Reports of tree hyrax hunting also exist from Mau Forest^18^ and from Côte d'Ivoire, where *Dendrohyrax dorsalis* is sold on bushmeat markets^47^. In the Udzungwa Mountains, tree hyraxes respond to hunting and other forms of human disturbance by reducing daytime call frequency, sun basking, and use of middens^28^. Tall upper canopy trees may protect animals from poaching, and canopy connectivity reduces the need to descend to the ground, which also reduces the risk of being trapped. We have been informed that in the Taita Hills, tree hyraxes have been hunted by knocking on trees where they have their sleeping cavities. As the animal is spooked, it jumps out and is killed if it loses its foothold and falls to the ground. It seems quite possible that the small tree hyrax populations in Chawia and Fururu, and those outside major forest fragments, are currently under poaching pressure, and that the populations would be larger and the animals more vocal if they were not disturbed.

Kundaeli^16^ described *Dendrohyrax validus* as a solitary forager with high intraspecific intolerance. We suggest that most animals in Taita Hills belong to groups that share common territories. We also suggest that active calling is used to keep contact with other group members, and to inform others that there is no danger at in sight, with some members of a group always on guard. Tree hyrax vocalizations in Taita Hills are extremely variable and graded^12^. For example, compared with the calling pattern of *D. arboreus* studied by Milner and Harris^19^, tree hyraxes in the Taita Hills are considerably more vocal. Their active calling and fast movements during cool nights also suggest that the animals have a relatively high metabolic rate, unlike other hyraxes^48,49^. In rock hyraxes (*Procavia capensis*), males produce loud and complex songs that convey multiple types of information about the singer^24–26,50,51^, and singing is related to mating behavior and social status of the singing male. By comparison, tree hyrax calling behavior has been much less studied and their songs were only first described by Rosti et al.^12^. Singing was most frequently recorded in the largest and least disturbed Mbololo, where tree hyraxes were singing during almost a quarter of all analyzed hours. This indicates that tree hyrax singing is affected by small population size and/or small and deteriorated habitat, and could thus potentially be used as an indicator of population viability. Unfortunately, we do not have information of the sex or social status of the singing individuals. We made our recordings in January, February, March, and September, and songs were heard and recorded during all these months. We heard singing most frequently heard in specific parts of the forests, which indicates that singing may be more frequent in some groups than in others.

In Taita Hills tree hyraxes only call during the night. Strictly nocturnal calling may be an adaptation to human disturbance^52^, but even after dusk, when tree hyraxes are already calling, local people often continue to move at edges of the studied forests. On the other hand, compared with animals in the three largest forest fragments, the tree hyraxes in Chawia and Fururu started their calling very late. The apparent shift in calling rate and pattern could be linked to reduced population density, or it could be a direct response to a high level of human disturbance, including poaching. In the Taita Hills, even the most isolated AudioMoth locations were within 600 m of farms or other buildings (average distance to a house was 143 m, median distance was 259 m), and all the forests are crisscrossed with paths that local people use for e.g. collecting firewood. The highest calling rate of tree hyraxes was recorded in the protected Ngangao Forest, where the most pristine parts of the forest had the most animals. The more isolated Mbololo Forest is also protected and had high tree hyrax calling rates. Vuria Forest is far more deteriorated and under considerable human pressure, but still supported a relatively dense population of tree hyraxes, which called actively from early evening onwards, sometimes very close to human dwellings. In some recordings, we could hear local people having parties or singing in a church while tree hyraxes were simultaneously having their own active calling bouts. On the contrary, Chawia seems to have very few calling tree hyraxes, and the small Fururu Forest was almost devoid of tree hyraxes. As forests become more disturbed, the height of trees tends to decrease and canopy connectivity is reduced, making hyraxes more vulnerable to poaching.

## Conclusions

Tree hyraxes depend on indigenous forest vegetation but seem to survive also in mixed forests if the size of the forest patch is large enough. In small and disturbed forests, tree hyrax density is reduced, their calling patterns change, and singing bouts become rare. Our results are generally consistent with a declining tree hyrax metapopulation within a highly fragmented forest landscape, mainly due to various forms of human disturbance. Protection of even small indigenous forest fragments, and particularly large trees and increasing connectivity among forest fragments is important for tree hyrax conservation, in conjunction with protecting the animals from poaching.

## Material and methods

We conducted field work during dry seasons 1 Jan–6 March and 5 Sept–1 Oct, in 2019–2021, in the five forests fragments characterized in Table S1. AudioMoth recordings were made between January and September. In addition to passive recordings, all forest fragments were also inventoried on foot to detect evidence of the presence of tree hyraxes (vocalizations, sightings of animals or their dung).

We used a thermal imaging camera, Pulsar Helion 2 XP50 (Yukon Advanced Optics Worldwide, Vilnius, Lithuania) and Fenix TK25 RED (Fenix Lighting, Littleton, CO, USA) and red beam flashlights, to find the animals in the forest canopy. Only red light was used. We took note of the tree hyraxes’ acoustic communication, feeding, movement patterns and social behavior. Observation times varied between 18:45 and 02:00 h or throughout the night. We could not always locate individuals in the higher canopy, even when we could hear their calls. When a tree hyrax was spotted, thermal camera recordings were made, and we pinpointed its GPS location with a Garmin Montana 610 (Garmin Ltd. Schaffhausen, Switzerland). For each observation, we noted the time, height in the canopy, distance from the observers, vocalizations, and whether the animal was feeding, resting or moving. Observation times of individual tree hyraxes ranged from only a few minutes to up to 1 h. Whenever possible, the animal was photographed, and its sex determined. We did not use this observational data to estimate population size, as visual sightings of tree hyraxes are random, and depend highly on wind conditions.

In Ngangao Forest, all occupied trees were marked during the night and identified to species in daylight (Table S2). Diameter at breast height (DBH) and canopy width were measured, and some phenological features (state of leaves and whether the tree was bearing fruit) were recorded. In Ngangao, we observed 155 individual tree hyraxes on 111 identified trees. In Mbololo Forest, trees were only identified from photographs taken during night. There a total of 20 photographs of tree hyraxes were taken, and 23 trees and climbers were identified from the images.

Tree hyrax distribution in the five forest fragments was studied using AudioMoth automatic recorders (v1.1.0 Open Acoustics Devices, Southampton, UK). 14 locations and 30 nights were included in the analysis. Differences in estimated tree hyrax population densities between sites were established by comparing calling rates/hour in different forests. Lidar data were used to determine and compare canopy structure and other stand characteristics at each AudioMoth site (Table S4). We expected tree hyrax population abundance to decrease with decreasing forest size, canopy height and canopy coverage.

The AudioMoth recorders were placed without preconceived bias into undisturbed locations in the central parts of each forest fragment. We used six recording sites from Mbololo, three sites from Ngangao, two sites from Vuria, two sites from Chawia, and one site from Fururu. We excluded nights with heavy rain as tree hyraxes were silent. In order to maintain data balance, we analyzed six nights from each forest (66 h per forest), except for Fururu, for which only five nights with recordings were available (55 h). Thus, a total of 319 recording hours were analyzed with the calling density calculated, for hours that tree hyraxes call actively, between 19:00 and 06:00 (Fig. 3, Table S3). An hour was marked as “tree hyrax singing present” if it contained at least five singing bouts including at least three different call types from crackle, rachet, wheeze, chuck and chirp, as defined in Rosti et al.^12^.

Recordings were analyzed with RAVEN PRO 1.6 (Cornell University, Ithaca, NY, USA) using the following spectrogram parameters: DFT size 512, 50% overlap, hann 86.1 Hz, sample rate 44,100 Hz, 16-bit signed. In Raven Pro Band Limited Energy Detector was used with the following parameters: minimum frequency 500 Hz, maximum frequency 4000 Hz, minimum duration 0.101 s, maximum duration 1 s, minimum separation 0.101 s, Signal-to-noise ratio minimum occupancy 70%. Every hour was inspected visually in a screen window of 1.1 min at a time. False positives that were caused by small-eared greater galago (*Otolemur garnettii*) calls or vocalizations of insects, frogs, dogs and birds, and other noise were removed from the data set.

We used airborne lidar (laser scanning) data to determine and compare canopy structure and other stand characteristics at each AudioMoth site (Table S4). Lidar data for the studied forests were acquired five years earlier than land survey in January–February 2014 and February 2015 using an aircraft-mounted Leica ALS60 sensor^53^. The mean flight altitude for the aircraft was approximately 1450 m above ground level, pulse rate 58 kHz, pulse rate 66 (Hz) and scan angle ± 16°. A maximum of four returns were recorded for each sent pulse. The resulting mean return density in the scanned area was 3.4 points per m^2^. Due to the time difference between lidar analysis (2014–2015) and land survey (2019–2021), some changes may have taken place in forest structure.

We classified lidar points to ground points and non-ground points and computed a digital terrain model and a canopy height model (CHM) at 1 m resolution. Furthermore, point clouds were normalized for the ground elevation to derive heights from the ground level. All this processing was made using tools available in the LAStools software (rapidlasso GmbH). For the analyses, we extracted normalized point clouds for circular areas of 0.5 ha (39.9 m radius) around AudioMoth sites. We used point clouds for calculating several metrics to characterize canopy structure (Table S4). Maximum canopy height was estimated using 99% percentile of the return heights. Maximum height was discarded as some sites included noise like points above canopy. Other canopy height metrics included mean of the return heights, and 75%, 50%, and 25% percentiles of return heights. Furthermore, variation in canopy height within the area was characterized by using the standard deviation and the coefficient of variation of return heights. For all these metrics, only returns above 3 m height were considered in order to separate ground/understory and canopy returns^53^. This threshold was selected because tree hyraxes require the presence of large upper canopy trees rather than small trees present in the understory. In addition, we also calculated several metrics related to canopy cover and density^54^ (Table S4). Canopy cover was defined as a ratio of the first returns from canopy to all first returns. Canopy density was defined as a ratio of all returns from canopy to all returns. Different thresholds were applied to define canopy returns including fixed 3 m, 10 m and 20 m heights, and mean and 75% canopy heights varying from one site to another. As all sites are situated in montane forest, they have high vegetation cover close to the ground (i.e. below 3 m) unless there are major gaps in the canopy. Canopy cover and density with larger thresholds, on the other hand, are sensitive to the presence of large, emergent trees, which can be important for tree hyraxes. All canopy structure metrics were calculated using lidR package^55^ in R environment^56^.

All statistical analyses were carried out in R studio^56^, with the packages “glmmTMB”^57^, and “performance”^58^. All graphs were created with the package “ggplot2”^59^. The modelling involved three different stages. Due to the high incidence of zero counts (24%), zero inflated negative binomial (ZINB) distribution was first employed to account for overdispersion of data caused by the excessive zeroes^60^. As these zeros came from only two forests (Chawia and Fururu), the other forests (Mbololo, Ngangao and Vuria) were also analyzed separately with negative binomial NB to enable analysis without zero inflation (as suggested by Campbell^61^). Generalized linear mixed models (GLMM) were implemented where the linear predictor contained random effect (site). This one-way nested model was used to ease dependency caused by using several hours from the same site (avoiding pseudoreplication). Continuous cofactors were standardized as required in the ZINB and NB GLMM and the glmmTMB package. Data exploration followed Zuur and Ieno^62^.

During analysis, the following variables were removed for collinearity: distance to forest edge, distance to nearest road and distance to nearest building, disturbance, zmean, zmax, zsd, zcv, cd10, cczmean, cdzmean, cczq75, cdzq75. An interaction between “forest size” and “time” was included in the model, as data exploration showed that tree hyraxes were calling at different times in forests of different sizes. Other covariates in the model were forest height (zq99) and canopy coverage (cc10) To evaluate model support, we assessed models based on the minimization of AIC (Tables 4B, 5B)^63^. Model assumptions were verified by plotting residuals versus fitted values, versus each covariate in the model and versus each covariate not in the model. We tested the model with zero inflation simulation^60^. Occurrence of tree hyrax songs were noted from each hour of the analyzed material by presence (1) or absence (0). The Bernoulli GLMM model was used to study effect of forest size in one-way nested model, with “site” as the random intercept. The model was kept simple as only 37 h contained singing. The model was validated by observation of residuals.

## Supplementary Information

Supplementary Information.

Supplementary Audio File S1.

## Data Availability

Dataset generated and analyzed during the current study is are available from the author (HR) upon reasonable request. Code is not available for copyright reasons.
