# Supplementary material for: Habitat preferences, estimated abundance and behavior of tree hyrax (Dendrohyrax sp.) in fragmented montane forests of Taita Hills, Kenya
Source: Sci Rep. 2022 Apr 15;12:6331. doi: 10.1038/s41598-022-10235-7 (PMC9012809; doi:10.1038/s41598-022-10235-7)
Supplement: Supplementary file 1 — Supplementary Information. [file 41598_2022_10235_MOESM1_ESM.pdf]

Habitat preferences, estimated abundance and behavior of tree hyrax (*Dendrohyrax* sp.) in fragmented montane forests of Taita Hills, Kenya.

Hanna Rosti, Janne Heiskanen, John Loehr, Henry Pihlström, Simon Bearder, Lucas Mwangala, Marianne Maghenda, Petri Pellikka, Jouko Rikkinen

Supplementary data

Supplementary Table S1. Description of the forest fragments studied. AudioMoth recordings were made in Mbololo, Ngangao, Vuria, Chawia, and Fururu Forests. Detailed behavioral observations were made in Ngangao and Mbololo. No tree hyraxes were detected in Kasigau and Sagalla.

**Mbololo**	Size 185 ha (3°19′37″ S, 38°27′4″ E), 1,550–1,700 m a.s.l. Well preserved and largely intact indigenous montane forest, only in places with patches of exotic plantation trees. The largest and least disturbed of the remaining indigenous forest fragments. Multi-layered canopy formed by many different tree species (Thijs 2015). The very steep slopes of Mbololo Ridge have helped to protect the forest from timber harvesting. Average canopy height at AudioMoth sites 27 m.
**Ngangao**	Size 120 ha (3°22′9″ S, 38°20′33″ E), 1,700–1,870 m a.s.l. Well preserved and largely intact indigenous montane forest, only in places with patches of exotic plantation trees. Multi-layered canopy formed by many different tree species, and with largest upper canopy trees over 50 m tall (Thijs 2015). Surrounded by agricultural land, and mostly easily accessible. Average canopy height at AudioMoth sites 34 m.
**Vuria**	Size 96 ha (3°25″ S, 38°17″ E), 1,900–2,200 m a.s.l. Heavily disturbed indigenous upper montane forest, in many places with intermixed exotic plantation trees. Average canopy height at AudioMoth sites 21 m.
**Chawia**	Size 85 ha (3°28″ S, 38°20″ E), 1,500–1,600 m a.s.l. Heavily disturbed indigenous montane forest in many places with intermixed exotic plantation trees, and with easy access from surrounding villages and agricultural land. Average canopy height at AudioMoth sites 33 m.
**Fururu**	Size 8 ha (3°25″ S, 38°20″ E), 1,650–1,750 m a.s.l. Severely disturbed indigenous montane forest, partly mixed with exotic plantation trees and with easy access from surrounding villages and agricultural land. Average canopy height ay AudioMoth sites 24 m.
**Kasigau**	Size 203 ha (3˚49’ S, 38˚40’E), 1,000–1,640 m a.s.l. Well-preserved and intact indigenous montane forest on the upper slopes of Mount Kasigau, with no exotic plantation trees. Multi-layered canopy formed by many different tree species (Meadley et al. 2015). Located 40 km southeast of the Taita Hills and separated from them by semiarid plains. Tree hyraxes were searched without success on foot and with AudioMoths placed in seven locations within the forest. Furthermore, according to interviews, local people living close to the forest edge have never heard their distinct calls.
**Sagalla**	Size 2 ha (03°30’S 38°35’E), 1,450-1,500 m a.s.l. Small remnant patch of indigenous forest on the upper E-facing slope of Sagalla Hill, surrounded by *Eucalyptus* plantations. Located 25 km to the southeast of the forest fragments on Dabida Massif and Mbololo Ridge. The forest was searched by foot (January 2021) without finding any evidence of the presence of tree hyraxes.

Supplementary Table S2. Indigenous broadleaved tree species with tree hyrax sightings in Ngangao Forest. NO = number of sightings. % = percentage of all sightings. DBH = average diameter of trunk (cm). DIAC = average diameter of canopy (m). Lianas = frequency of lianas around the trunks (%).

Tree species	NO	%	DBH	DIAC	Lianas
Macaranga capensis	23	21.9	48.1	10.1	47.8
Tabernaemontana stapfiana	22	21.0	43.0	7.5	40.9
Albizia gummifera	14	13.3	73.5	14.6	50
Strombosia scheffleri	7	6.7	58.9	10.1	42.9
Syzygium cordatum	7	6.7	89.1	14.1	71.4
Celtis africana	6	5.7	23.9	7.5	83.3
Vitex keniensis	6	5.7	49.3	10.5	33.3
Millettia oblata	4	3.8	74.5	13.8	25
Pouteria adolfi-friedericii	4	3.8	138.1	12.7	75
Cola greenwayi	2	1.9	30.7	8.3	50
Englerophytum natalense	2	1.9	80.2	14.4	100
Ochna holstii	2	1.9	41.4	11.2	100
Maesa lanceolata	1	1	76.4	10.6	0
Newtonia buchananii	1	1	53.2	8.5	0
Nuxia floribunda	1	1	70.0	7.5	0
Ocotea usambarensis	1	1	76.0	10.6	100
Polyscias stuhlmannii	1	1	29.9	7.3	0
Rapanea melanophloeos	1	1	64.3	13.5	0

Supplementary Table S3. *Dendrohyrax* vocalizations recorded from five forest fragments in the Taita Hills. AV = average number of recorded tree hyrax calls per hour, with standard deviation (SD) and median (MED). Singing = total number of hours with tree hyrax singing, with percentage of all recorded hours (%).

	Mbololo	Ngangao	Vuria	Chawia	Fururu
**AV**	89	116	66	12	3
**SD**	64.7	95.4	43.2	20.4	8.7
**MED**	80.5	81.5	53	0	0
**Singing**	15	14	6	1	1
**%**	23	21	9	1.5	1.8

Supplementary Table S4. Variables analyzed from AudioMoth study sites. Lidar variables were determined from circular areas of 0.5 ha (39.9 m radius) around each AudioMoth recorder.

Variable	Description
calls	Response variable, number of recorded tree hyrax calls per hour, AudioMoth.
time	Each hour (19–06) analyzed separately (19:00–19:59=1, 20:00–20:59=2, etc.).
song	Presence or absence (0/1) of tree hyrax songs during focal hour, AudioMoth.
size	Size of forest fragment (ha).
distance to edge	Distance from AudioMoth site to nearest forest edge (m), lidar.
distance to building	Distance from AudioMoth site to nearest building (m), lidar.
distance to road	Distance from AudioMoth site to nearest road (m), lidar.
elevation	Elevation (m.a.s.l.) of the AudioMoth site (from GPS).
zmean	Mean canopy return height (only returns >3 m in height), lidar.
zmax	Maximum canopy return height (only returns >3 m in height), lidar.
zq99	99% percentile return height (only returns >3 m in height), lidar.
zsd	Standard deviation of canopy return height (only returns >3 m in height), lidar.
zcv	Coefficient variation of canopy return height (only returns >3 m in height), lidar.
cc10	Canopy cover (10 m height threshold), lidar.
cd10	Canopy density (10 m height threshold), lidar.
cczmean	Canopy cover at mean canopy return height, lidar.
cdzmean	Canopy density at mean canopy return height, lidar.
cczq75	Canopy cover at 75% percentile canopy return height, lidar.
cdzq75	Canopy density at 75% percentile canopy return height, lidar.
disturbance	Relative disturbance: 1 (disturbed) – 5 (pristine), estimated in the field.

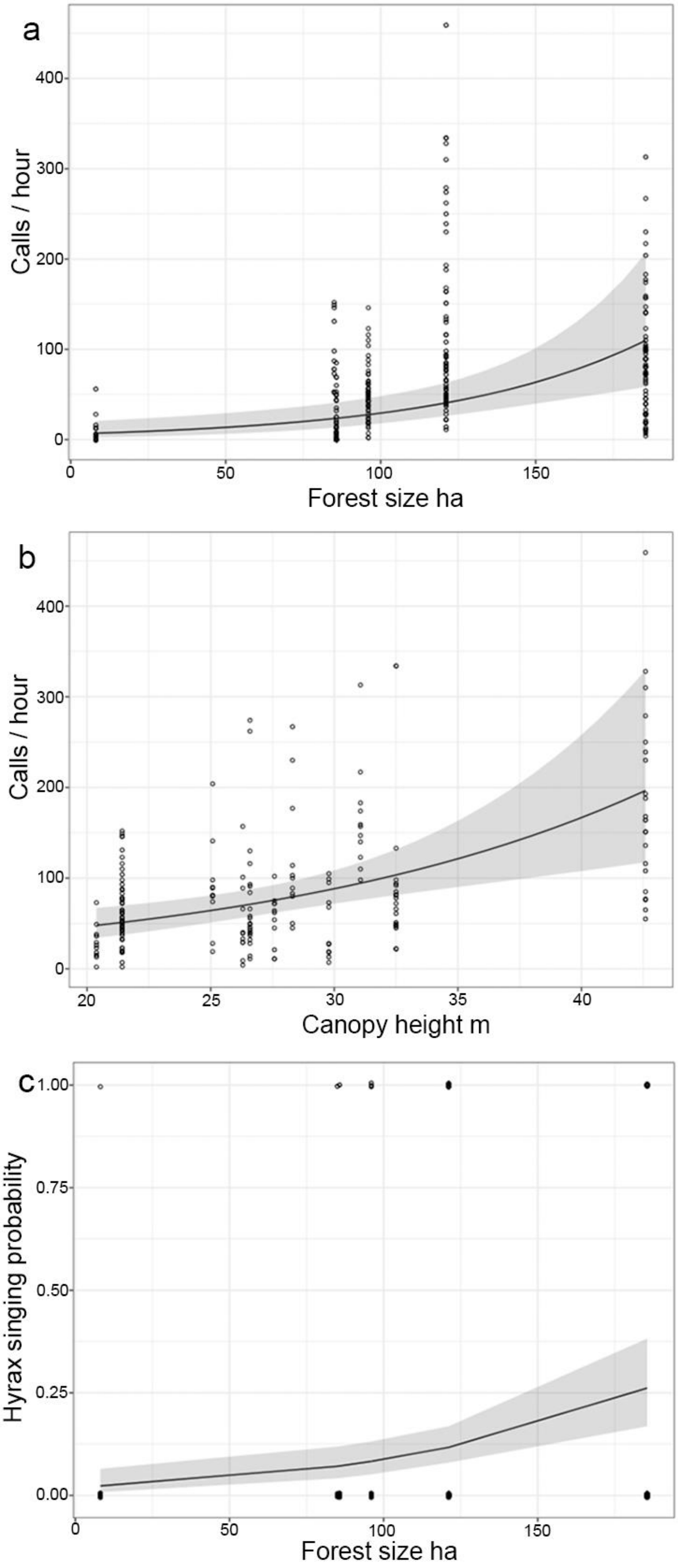

Supplementary Figure S1. Results of GLMM analysis. **a** ZINB GLMM model for all forest fragments indicting that forest size is a significant determinant of calling frequency. **b** NB GLMM model for the largest forest fragments Mbololo, Ngangao and Vuria (forests without zero inflation) indicating that canopy height is a significant determinant of calling frequency. **c** Bernoulli GLMM model for all forest fragments indicating that forest size is a significant determinant of singing probability. The graphs were created with software R version 3.6.3, https://www.r-project.org/", and modified with Adobe Photoshop, version 22.3, https://www.adobe.com/.

Supplementary Audio File S1. Sample of tree hyrax song (Mbololo Forest 2021).

Audio file S1.wav
